# Hospital use of common Z-codes for Medicare fee-for-service beneficiaries, 2017–2021

**DOI:** 10.1093/haschl/qxad086

**Published:** 2023-12-14

**Authors:** Ji E Chang, Nate Smith, Zoe Lindenfeld, William B Weeks

**Affiliations:** Department of Public Health Policy and Management, New York University School of Global Public Health, New York, NY 10003, United States; CareJourney, Arlington, VA 22203, United States; Department of Public Health Policy and Management, New York University School of Global Public Health, New York, NY 10003, United States; AI for Good Lab, Microsoft Corporation, Redmond, WA 98052, United States

**Keywords:** social determinants of health, screening, hospitals, community health

## Abstract

Recognizing the impact of the social determinants of health (SDOH) on health outcomes, in 2016, the Centers for Medicare and Medicaid Services recommended the use of *International Classification of Diseases, 10th Revision* (ICD-10), Z-codes to capture patients' health-related social needs. We examined changes in Z-code utilization to document health-related social needs for Medicare fee-for-service recipients among US hospitals between 2017 and 2021 across 5 common SDOH domains. We found that, while 56.9% of hospitals had at least 1 Z-code recorded in at least 1 patient per year, apart from those referring to housing needs, rates of Z-code adoption were low. Additionally, hospitals that were general medical, part of a teaching institution, affiliated with larger health systems, and of medium to large size had greater odds of utilizing Z-codes. Findings from this study highlight the need for continued efforts in promoting the consistent use of standardized SDOH capturing methods like Z-code documentation, such as provider training.

## Introduction

The social determinants of health (SDOH), defined as the “conditions where individuals are born, grow, work, live, and age,” play a pivotal role in shaping health outcomes.^1^ Recognizing this link, key regulatory and standard-setting agencies have introduced quality measures to foster health equity by gathering patient health-related social needs (HRSN) data.^2,3^ Notably, starting in 2024, the Centers for Medicare and Medicaid Services (CMS) will mandate HRSN screening in 5 common SDOH domains (housing, food, transportation, utilities, and interpersonal safety) during adult hospitalizations.^4^ In this context, *International Classification of Diseases, 10th Revision* (ICD-10), Z-codes that capture nonmedical determinants of health provide a standardized method for documenting and sharing SDOH across health care entities. However, their adoption has faced challenges, slowing widespread use.

**Figure 1.**
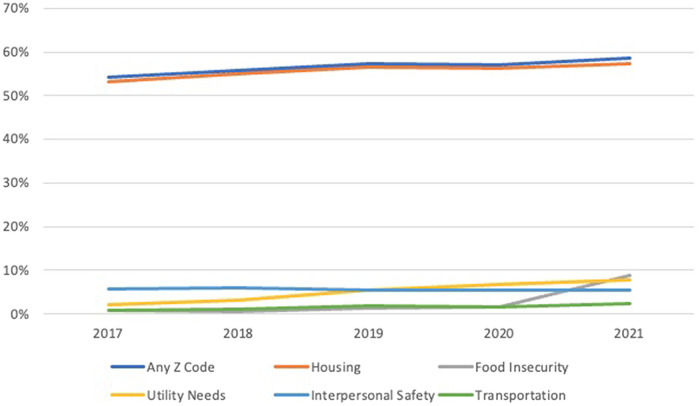
Percentage of hospitals with at least 1 Z-code recorded, overall and in each category, per year (2017–2021). Medicare fee-for-service claims data for 42 million patients were aggregated at the hospital level.

We sought to examine change in the utilization of Z-codes in Medicare fee-for-service (FFS) hospitalizations in US hospitals from 2017 to 2021 by SDOH domain (Figure 1). Numerous studies have examined patient characteristics associated with Z-code use.^5-7^ However, less is known about facility and community characteristics associated with Z-code adoption. While a few studies included facility characteristics in their analysis, they drew from pediatric patient data or are limited to a single state or setting.^8-13^ We extend this work by examining facility and community characteristics associated with Z-code utilization across US hospitals across the 5 HRSN screening domains slated to be tracked by CMS, with a goal of providing insights into institutional practices, policies, and resources, which are pivotal for the systematic integration of SDOH data in hospitals that will be required in upcoming regulatory requirements surrounding HRSN screening and data collection.^4^

## Data and methods

Our analysis of the adoption of Z-codes in the United States combines data from the American Hospital Association (AHA) Annual Survey with total Medicare FFS claims from 42 million patients aggregated at the hospital level using the CareJourney analytic platform.^14^ The AHA survey annually captures data from over 6000 US hospitals and health care systems. Our analytic sample comprises 27 385 observations from 5685 hospitals over 5 years, encompassing all nonfederal, nonpediatric, acute-care hospitals processing at least 1 Medicare FFS claim annually.

We formed binary variables corresponding to each of the 5 HRSN categories (housing, food, transportation, utilities, and interpersonal safety) based on specific Z-code descriptions from the CMS (Table 1) and determined whether each hospital used at least 1 Z-code in any category, or in each specific category, for at least 1 patient annually (Table 2). Hospital features, such as teaching status, system membership, critical access designation (CAH), ownership, bed size, and region, were sourced from the AHA survey. County attributes were derived from the American Community Survey (percent uninsured, percent Black, percent Hispanic/Latinx, percent unemployed, percent high school education), the US Department of Agriculture (metropolitan status), and the University of Wisconsin's Area Deprivation Index. To connect claims measures to the AHA survey, we matched CMS Certification Number and Hospital Name identifiers to the AHA Hospital ID. County data were linked using Federal Information Processing Standards (FIPS) codes.

**Table 1.** Social determinants of health domains and Z-code cross-walk.

SDOH domain	Z-code
Housing (instability, quality, financing)	Z59.0 Homelessness Z59.1 Inadequate housing Z59.8 Other problems related to housing and economic circumstances Z59.9 Problem related to housing and economic circumstances, unspecified
Food insecurity or hunger	Z59.4 Lack of adequate food and safe drinking water
Utility needs	Z59.7 Insufficient social insurance and welfare support Z58 Problems related to physical environment
Interpersonal [safety] violence	Z65.4 Victim of crime and terrorism Z65.8 Other specified problems related to psychosocial circumstances
Transportation	Z59.2 Other problems related to housing and economic circumstances including transportation insecurity

Source: Centers for Disease Control and Prevention Comprehensive Listing, ICD-10-CM files.

Abbreviations: ICD-10-CM, *International Classification of Diseases, 10th Revision–Clinical Modification*; SDOH, social determinants of health.

**Table 2.** Percentage of hospitals with at least 1 Z-code recorded per year (2017–2021).

	2017	2018	2019	2020	2021	Mean	Change^a^
Any Z-code	54.5%	56.4%	57.3%	57.4%	59.1%	56.9	+4.6%
Housing	53.7%	55.4%	56.6%	56.2%	57.7%	56.0	+4.0%
Food insecurity	0.8%	0.7%	1.4%	1.6%	8.9%	2.6	+8.1%
Utility needs	2.1%	3.2%	5.4%	6.9%	7.9%	5.0	+5.8%
Interpersonal safety	5.8%	6.1%	5.5%	5.5%	5.5%	5.7	−0.3%
Transportation	0.8%	1.0%	1.8%	1.8%	2.4%	1.6	+1.6%

Medicare fee-for-service claims data for 42 million patients aggregated at the hospital level.

^a^Describes change from 2017 to 2021.

We calculated mean hospital and county characteristics across all observations (Table 3). We used generalized estimating equations for longitudinal data to generate unadjusted and adjusted odds ratios (ORs) (with 95% CIs) estimating relationships between hospital-level adoption of Z-codes, year, hospital characteristics, and county characteristics (Table 4). All analyses were conducted using STATA 16.0 SE (StataCorp LLC). This study of de-identified hospital-level data was exempt from the New York University Institutional Review Board.

**Table 3.** Descriptive statistics of hospitals, 2017–2021.

	Values, %
Hospital characteristics	
General medical	77.2
Teaching hospital	39.6
System membership	66.7
Critical access	23.9
Ownership	
Nonprofit	53.1
Public	20.3
For profit	26.6
Bed number (hospital size)	
<50 (small)	36.9
50–199 (medium)	38.4
200+ (large)	24.8
County characteristics	
Black	11.6
Hispanic/Latinx	14.0
Unemployed	5.2
High school graduate	29.8
Uninsured	11.3
Area Deprivation Index (mean)	42.7
Metropolitan	65.9

*n* = 27 385 observations across 5685 hospitals. Hospital characteristics were derived from the American Hospital Association (AHA) survey.

**Table 4.** Unadjusted and adjusted odds ratios generated from generalized estimating equation models of hospital and community characteristics associated with any Z-code use.

	Unadjusted models		Adjusted models	
	OR	95% CI	OR	95% CI
Year	1.04***	(1.03-1.05)	1.05***	(1.03-1.07)
Hospital characteristics				
Specialty hospital	Ref		Ref	
General medical	3.00***	(2.70-3.33)	6.98***	(6.11-7.97)
Teaching hospital	2.43***	(2.27-2.60)	1.45***	(1.32-1.60)
System membership	2.23***	(2.05-2.41)	1.72***	(1.56-1.91)
Critical access	0.13***	(0.12-0.15)	0.25***	(0.22-0.29)
Ownership				
Nonprofit	2.02***	(1.97-2.19)	Ref	Ref
Public	0.45***	(0.41-0.50)	0.60***	(0.53-0.69)
For profit	0.80***	(0.73-0.88)	0.92	(0.81-1.04)
Bed number (hospital size)				
<50 (small)	0.17***	(0.15-0.18)	Ref	
50–199 (medium)	1.42***	(1.31-1.52)	2.44***	(2.20-2.71)
200+ (large)	7.45***	(6.59-8.43)	7.36***	(6.17-8.78)
County characteristics				
% Black^a^	1.08***	(1.05-1.12)	0.92***	(0.88-0.96)
% Hispanic/Latinx^a^	1.21***	(1.17-1.25)	1.08***	(1.04-1.13)
% Unemployed^a^	1.31***	(1.18-1.48)	1.74***	(1.45-2.09)
% High school^a^	0.47***	(0.45-0.50)	0.66***	(0.61-0.73)
% Uninsured^a^	0.70***	(0.65-0.75)	0.84**	(0.97-1.03)
Area Deprivation Index^a^	0.90***	(0.89-0.91)	1.00	(0.97-1.03)
Metropolitan	3.69***	(3.35-4.06)	1.40***	(1.22-1.61)

*n* = 27 385 observations across 5685 hospitals. Hospital characteristics were derived from the American Hospital Association (AHA) survey. Percent uninsured was drawn from the American Community Survey, metropolitan status was derived from the Area Health Resource File, and the Area Deprivation Index from the University of Wisconsin. Z-code documentation was drawn from Medicare fee-for-service claims data for 42 million patients aggregated at the hospital level. ***P < .001; **P < .01.

Abbreviations: OR, odds ratio; Ref, referent.

^a^Per 10% change.

## Results

Our analysis of hospital-based Z-code data found that, on average, 56.9% of hospitals had at least 1 Z-code recorded each year between 2017 and 2021. However, with the exception of housing, rates of Z-code documentation were low. While more than half of the hospitals in our sample recorded Z-codes relating to housing (56.0%), less than 10% documented a single Z-code on a single patient relating to food insecurity (2.6%), utility needs (5.0%), interpersonal safety (5.7%), or transportation (1.6%). For most Z-code categories, documentation increased over time, with the largest increase occurring for food insecurity (from 0.8% of hospitals in 2017 to 8.8% of hospitals in 2021). Only Z-code documentation for interpersonal safety decreased (from 5.8% of hospitals in 2017 to 5.5% of hospitals in 2021) (Table 2).

Most hospitals in our sample were general medical (77.3%). Approximately two-fifths were part of teaching hospitals (39.6%) and two-thirds were part of a larger health care system (66.7%). Approximately one-quarter of hospitals in our sample were CAHs (23.9%), and approximately half were nonprofit hospitals (53.1%). Most hospitals were small (36.5%) or medium-sized (38.4%) (Table 3).

In longitudinal data analysis, a number of hospital and county characteristics were significantly associated with the utilization of any Z-codes (Table 4). Specifically, general medical hospitals had significantly higher odds (adjusted OR [aOR], 6.98; 95% CI, 6.11–7.97) of Z-code utilization compared with specialty hospitals. Teaching hospitals (aOR, 1.45; 95% CI, 1.32–1.60), hospitals that belonged to systems (aOR, 1.72; 95% CI, 1.56–1.91), and medium (aOR, 2.44; 95% CI, 2.20–2.71) and large (aOR, 7.36; 95% CI, 6.17–8.78) hospitals had significantly higher odds of utilizing Z-codes than their counterparts. Meanwhile, CAHs (aOR, 0.25; 95% CI, 0.22–0.29) and public hospitals (aOR, 0.60; 95% CI, 0.53–0.69) had significantly lower odds of utilizing Z-codes compared with non-CAHs and nonprofit hospitals, respectively. The proportion of African-Americans in the surrounding county was negatively associated with hospital adoption of Z-codes (aOR, 0.92; 95% CI, 0.88–0.96), while the opposite was true for that of the Hispanic/Latinx population (aOR, 1.08; 95% CI, 1.04-1.13). Local unemployment rates (aOR, 1.74; 95% CI, 1.45–2.09) and metropolitan status (aOR, 1.4; 95% CI, 1.22–1.61) were positively associated with Z-code utilization, while high school graduation (aOR, 0.66; 95% CI, 0.61–0.73) and uninsured rates (aOR, 0.84; 95% CI, 0.97–1.03) were negatively associated with the use of Z-codes in hospitals.

## Discussion

Z-code documentation is being encouraged by CMS for risk-adjustment and facilitating consistent comparisons and analyses of SDOH across health care settings. Despite this encouragement, we found a low rate of Z-code adoption across most domains, with the relatively higher documentation of housing Z-codes that we found being consistent with prior literature.^8,15^ Importantly, over the span of our study, there was merely a marginal uptick in Z-code adoption. This trend underscores a persistently low inclination towards Z-code documentation among health care providers, despite its evident significance. Given the unfolding of the COVID-19 pandemic during our study period, one might hypothesize an accelerated recognition of the importance of SDOH, since SDOH were related to COVID-19 outcomes. However, our findings suggest that, even in the face of such a significant global health crisis, the adoption rate of Z-codes remained stubbornly low. This raises questions on the preparedness and emphasis that health care settings place on holistic understanding of SDOH. To bridge this gap, hospitals may benefit from comprehensive training programs that emphasize the importance of all SDOH domains and for establishing robust systems and partnerships that can address the myriad of nonmedical needs patients may present with beyond housing.

Our study sheds light on specific facility characteristics influencing the utilization of Z-codes to document HRSN for Medicare FFS patients. Hospitals that were general medical, part of a teaching institution, affiliated with larger health systems, and of medium to large size had greater odds of utilizing Z-codes. It is plausible that these hospitals, given their larger infrastructure and resources, might be better positioned to integrate new workflows required to screen for HRSN and document need using Z-codes.^11^ Conversely, CAH and public hospitals have decreased odds of such utilization; given the pivotal role these hospitals play in serving more vulnerable populations, it is important to address the barriers they face in regularly documenting Z-codes.

There are several limitations to our study. First, our study is restricted to Medicare FFS claims and excludes other payers. Considering the influence of other value-based care incentive programs in Medicaid and managed Medicare on the documentation of social needs, our dataset might not fully capture the extent of Z-code utilization across hospitals. This is particularly true if these codes are documented more frequently among patients not covered by Medicare FFS. However, our metric for Z-code adoption—represented by hospitals with at least 1 Medicare FFS claim noting a Z-code—is a relatively conservative measure, setting a minimal threshold for documentation, the analysis of which might generate different results. Second, hospitals may be documenting HRSN in their electronic health records systems but not fully converting these needs into Z-codes for claims for billing purposes.^16^ Nonetheless, to the extent that CMS uses billing coding to calculate risk scores, those hospitals may miss reimbursement opportunities. Second, while we have captured patterns of Z-code capture at the county level, our hospital-based analysis does not incorporate individual patient characteristics.^8^ Third, our findings are constrained by the data available up to 2021, potentially missing recent shifts in practices or policies following COVID-19. Finally, our measure of Z-code documentation—at least 1 Z-code documented for at least 1 patient per year—presented a very low bar for “documentation.” For Z-codes to be effectively used, a substantial proportion of patients will need to have documentation.

Despite these limitations, our study offers insights into the current state of Z-code documentation across US hospitals among Medicare FFS beneficiaries and highlights the need for more consistent and comprehensive documentation of SDOH. Findings from this study highlight the need for continued efforts in promoting the consistent use of standardized SDOH collection tools like Z-codes. Future research should explore the mechanisms and barriers faced by hospitals in integrating Z-codes into their billing and documentation systems.

## Supplementary Material

qxad086_Supplementary_Data
